# Optimal woven endobridge (WEB) device-aneurysm volume: using automated volumetric software to predict aneurysmal occlusion status after WEB treatment

**DOI:** 10.1007/s00701-025-06558-9

**Published:** 2025-09-13

**Authors:** Yosuke Kawamura, Tomoji Takigawa, Masaya Nagaishi, Akio Hyodo, Kensuke Suzuki

**Affiliations:** 1 Department of Neurosurgery, Dokkyo Medical University Saitama Medical Center, 2-1-50 Minami Koshigaya, Koshigaya Shi, Saitama, 343-8555 Japan; 2 Department of Neuroendovascular, Kamagaya General Hospital, Chiba, Japan

**Keywords:** Endovascular therapy, Intracranial aneurysm, Woven EndoBridge, Three-dimensional software, Wide-neck bifurcation aneurysms, Occlusion prediction

## Abstract

**Background:**

Optimal Woven EndoBridge (WEB) sizing is crucial for successful implantation. We used three-dimensional (3D) software, Siemens Aneurysm Analysis v. VD30B, to determine the potential of WEB device-aneurysm volume (DAV) ratios in predicting post-treatment occlusion status.

**Methods:**

We retrospectively reviewed 35 unruptured intracranial wide-neck bifurcation aneurysms treated using the WEB. The study evaluated the association between the DAV ratio and aneurysm obliteration during follow-up. The primary aim of the study was to determine the optimal DAV ratio for predicting occlusion status following WEB treatment.

**Results:**

The success rate of treating all unruptured aneurysms with the WEB was 100%. The median duration to final follow-up was 365 ± 184.0 days with all 35 patients having a mean modified Rankin Scale score of 0. One (2.9%) patient experienced a transient ischemic attack with hemiparesis perioperatively. A follow-up angiography revealed complete occlusion (WEB Occlusion Scale [WOS] A and B) in 25 (71.4%) of 35 patients and WOS C and D in 10 (28.6%) of 35. The median DAV ratio significantly differed between the groups. The median DAV ratios were 0.97 in the complete occlusion group and 0.84 in the WOS C and D group, respectively (*p* = 0.002). A receiver operating characteristic curve produced an area under the curve of 0.76 (confidence interval: 0.56–0.96). The optimal DAV ratio cut-off and the highest Youden index for complete occlusion was 0.90.

**Conclusion:**

Calculating DAV ratios using 3D software may help improve the rate of complete occlusion following WEB treatment.

## Introduction

Endovascular treatment of wide-neck bifurcation aneurysms (WNBAs) is challenging because it requires embolizing aneurysms while preserving bifurcated vessels to achieve complete occlusion [11]. The Woven EndoBridge (WEB, MicroVention, Inc., Aliso Viejo, CA, USA) has established intrasaccular flow disruption as a reliable treatment option for a wide range of intracranial aneurysms including WNBAs [10].

Selecting the optimal size of a WEB device is essential for successful implantation. The conventional recommendation for WEB sizing is based on the + 1/−1 rule or a size chart provided by the manufacturer based on the width and height of aneurysms [16]. Three-dimensional (3D) digital subtraction angiography (DSA) reconstruction and/or two-dimensional (2D) DSA presently determine the ideal size of WEB devices [11]. Manual measurement alone may be insufficient for selecting the optimal WEB size. The WEB device-aneurysm volume (DAV) ratio, defined as the volume of the WEB device divided by the volume of the aneurysm, offers a more precise assessment. [3, 16]

Using the DAV ratio and 3D Aneurysm Analysis (Siemens Healthineers, Forchheim, Germany) might improve the success rate of WEB device implantation compared with using manual, 3D or 2D DSA to determine the dimensions of aneurysms. The rate of implant success was the highest within a DAV ratio range of 0.6‒0.8 [3]. The ideal WEB-aneurysm volume (iWAVe) ratio is a novel, volume-based parameter for optimal WEB sizing. Aneurysms treated regardless of correct or incorrect WEB size were evaluated to determine the iWAVe ratio. Logistic regression analysis associated an iWAVe ratio of 0.90‒1.16 is with > 80% probability of correct sizing, as indicated by the 95% lower confidence interval (CI) [16]. These ratios indicated successful implantation. However, the optimal size selection for predicting complete occlusion after WEB treatment is not guaranteed. This study aimed to determine the optimal DAV ratio for predicting occlusion status after WEB treatment.

## Methods and materials

### Patient selection and data collection

We retrospectively explored our internal database for all patients with intracranial unruptured WNBAs treated with WEB between February 2021 and August 2024. Patients treated with WEB SL were included. We defined WNBAs as bifurcation aneurysms with a neck width of ≥ 4 mm and a dome-to-neck ratio < 2. We analyzed baseline patient demographic data, aneurysm characteristics, procedural variables, and aneurysm occlusion status at the final follow-up. Aneurysm characteristics comprised height, width, depth, neck, and volume.

#### Aneurysm measurements

WEB size was initially determined based on the average width and height of two projections using manual 3D DSA. The strategy involved selecting implants that were + 1 or + 2 mm wider than the aneurysm width and –1 or –2 mm shorter than its height [3, 10].

**Fig. 1:**
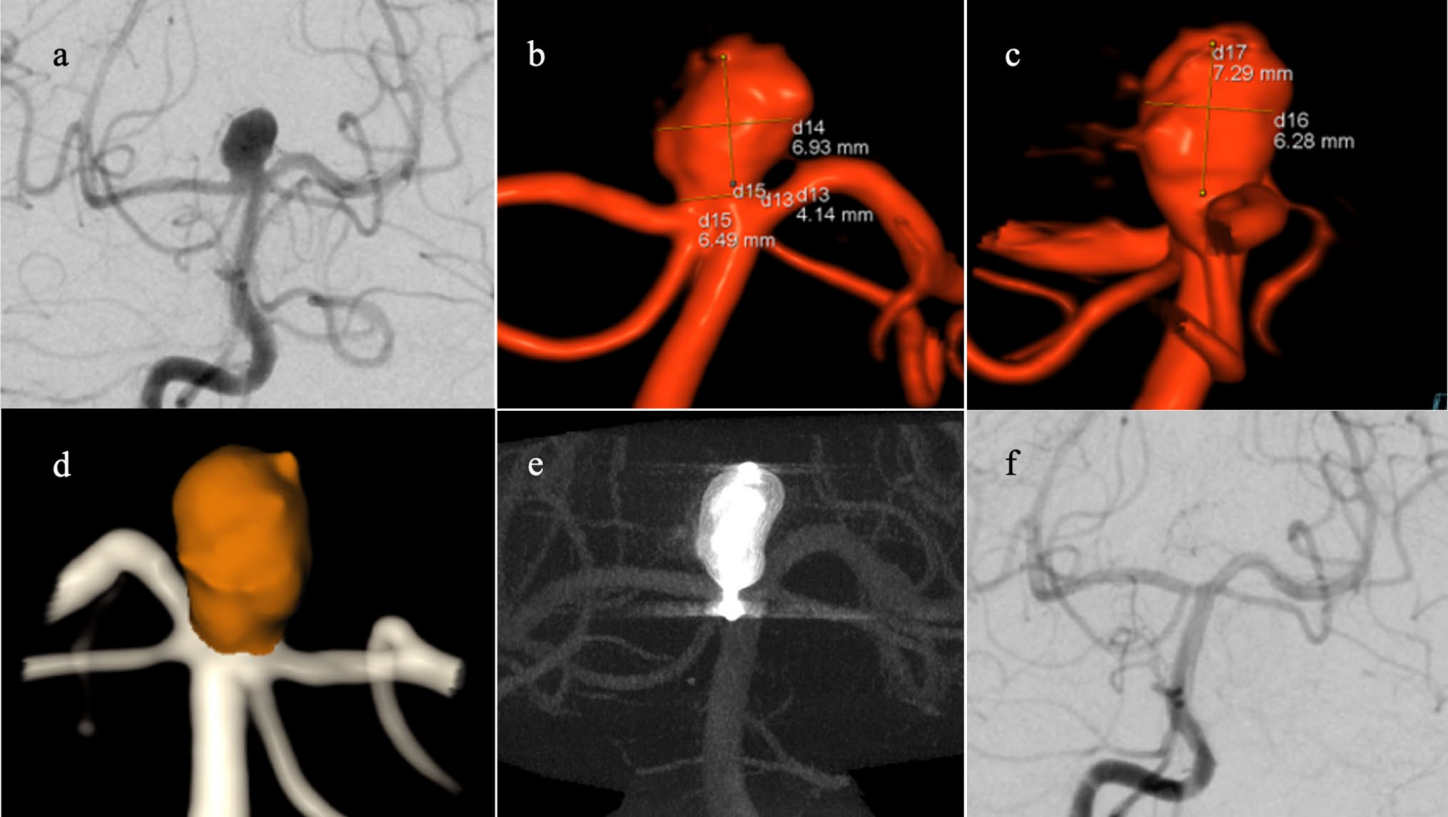
Angiographic measurement modalities. **a** Manual two-dimensional (2D) anteroposterior angiogram of basilar top aneurysm. **b** Three-dimensional (3D) angiographic reconstruction (anteroposterior view) shows manual measurements of aneurysmal height, width, and neck. **c** 3D angiographic reconstruction (lateral view) with manual measurements of aneurysmal height and width. **d** Automatic analysis and calculation of aneurysmal volume (248 mm^3^). **e** Multiplanar reconstructed dynamic computed tomography image acquired after injecting dilute contrast, shows deployed Woven EndoBridge (WEB) SL 8 × 5 (WEB volume, 251 mm^3^; Device-aneurysm volume ratio, 1.01). **f** Image at 6 months after WEB deployment shows complete occlusion, graded as WEB Occlusion Scale (WOS) A

**Fig. 2:**
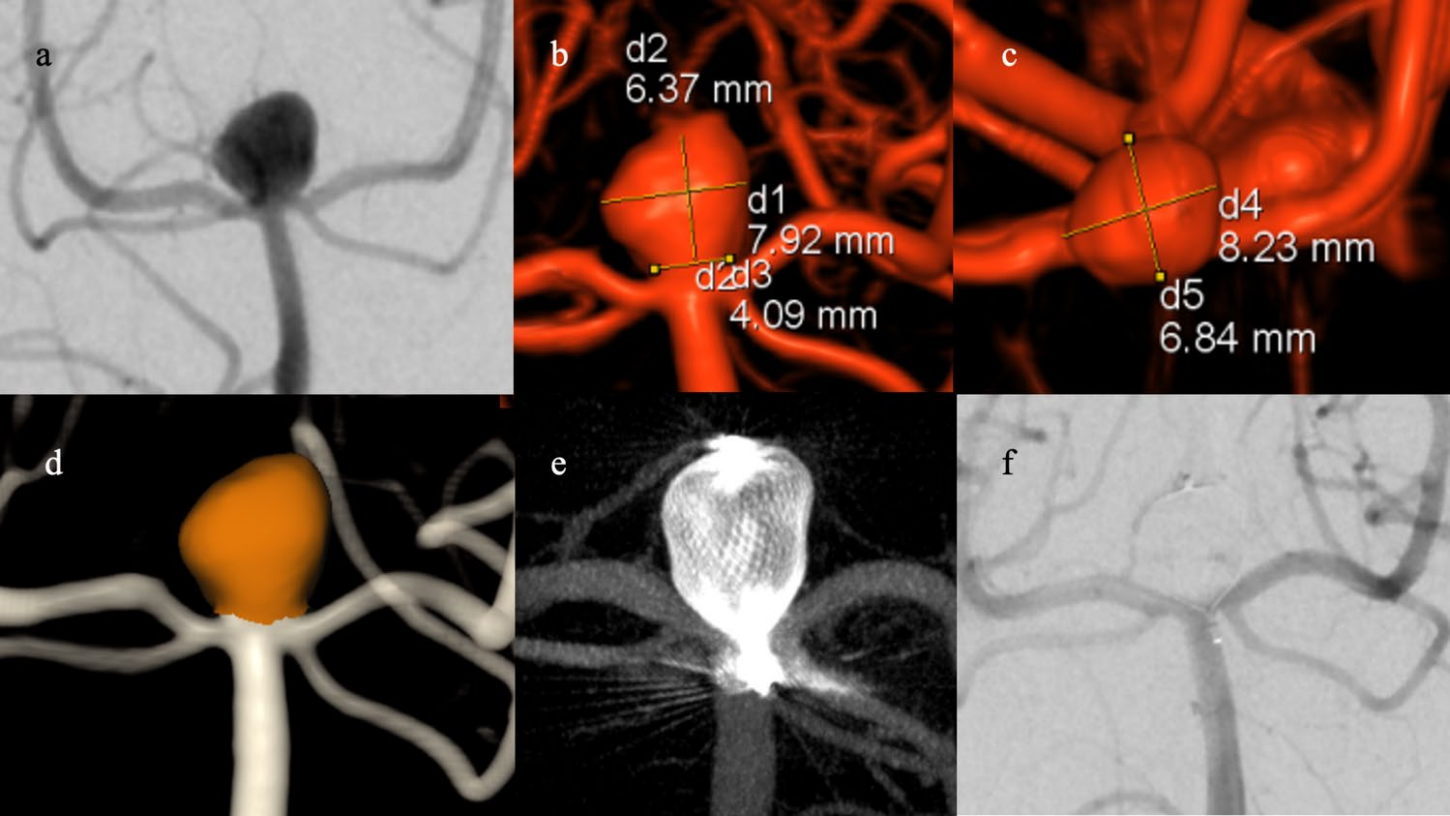
Angiographic measurement modalities. **a** Manual 2D anteroposterior angiogram of basilar top aneurysm. **b** 3D angiographic reconstruction (anteroposterior view) shows manual measurements of aneurysmal height, width, and neck. **c** 3D angiographic reconstruction (cranial-caudal view) with manual measurements of aneurysmal width. **d** Automatic analysis and calculation of aneurysmal volume (273 mm^3^). **e** Multiplanar reconstructed dynamic computed tomography image acquired after injecting dilute contrast, shows deployed WEB SL 9 × 4 (WEB volume, 254 mm^3^; Device-aneurysm volume ratio, 0.93). **f** Image at 6 months after WEB deployment shows complete occlusion, graded as WOS A

Subsequently, 3D DSA aneurysmal volumes were calculated before WEB treatment using Aneurysm Analysis v. VD30B (Siemens Healthineers, Forchheim, Germany) after placing seed pixels: one point in the center of the aneurysm sac and two in the parent vessel. We chose this software as it is allegedly more accurate than other automated modalities [7, 16]. Figures 1 and 2 show the manual 3D measurements and the automated aneurysmal volume.

#### Device-aneurysm volume ratio

The DAV ratio was defined as the volume of the WEB device divided by the aneurysm volume. The volume of the WEB device was determined based on the manufacturer’s sizing chart. The DAV ratio was calculated to evaluate its association with aneurysm obliteration at follow-up [16].

#### Procedural details

Patients were treated with oral aspirin (100 mg/day) and clopidogrel (75 mg/day) for 2 weeks before endovascular treatment. Aspirin and P2Y12 reaction units were measured using rapid platelet function assays (Verify Now; Accumetrics, Inc., San Diego, CA, USA) to assess resistance to aspirin or clopidogrel. The aspirin dose was increased or prasugrel 3.75 mg/day was prescribed if resistance occurred. Clopidogrel and prasugrel were discontinued immediately after the procedure, whereas aspirin was continued for 3 months after treatment.

A VIA™ catheter (MicroVention, Tustin, CA, USA) was navigated into the aneurysms typically by unsheathing until it formed a “flower” that pushed a microwire to deploy the WEB. The extent of WEB protrusion into the parent artery was then assessed by angiography and cone-beam computed tomography with diluted contrast medium. Occasional re-sheathing and repeated deployment proceeded as needed. The WEB device was subsequently detached.

#### Radiological assessment

The primary outcome was determining the association of the DAV ratio with aneurysm obliteration at follow-up. Four-vessel DSA was conducted at 3- or 6-month follow-up visits. Some patients underwent DSA at 12 months if the aneurysm was not occluded at 6 months. Occlusion on follow-up DSA was graded according to the WEB Occlusion Scale (WOS). Complete occlusion was classified as WOS grade A (complete occlusion) or B (complete occlusion with a marker recess). Residual neck filling (C) was indicated by contrast opacification of the aneurysm neck extending beyond the expected bounds of the marker recess. Residual aneurysm filling (D) was indicated by contrast opacification extending beyond the aneurysm neck into the fundus [8]. The WEB-IT study defines adequate occlusion as complete or neck remnants [9]. We compared the completely occluded WOS A and B, with the C and D groups.

#### Statistical analysis

Our dataset consisted of continuous and categorical variables. Between-group differences were compared using t-tests or Mann–Whitney U tests for continuous variables, and Fisher exact tests for categorical variables. The sensitivity and specificity of the DAV ratio compared with completely occluded and WOS C and D were analyzed at follow-up by estimating the area under the receiver operating characteristic (ROC) curve (AUC). Cut-off optimal DAV ratios were calculated using the Youden index. All data were statistically analyzed using SPSS 29 (IBM Corp., Armonk, NY, USA). Values with *p* < 0.05 were considered statistically significant.

## Results

**Fig. 3:**
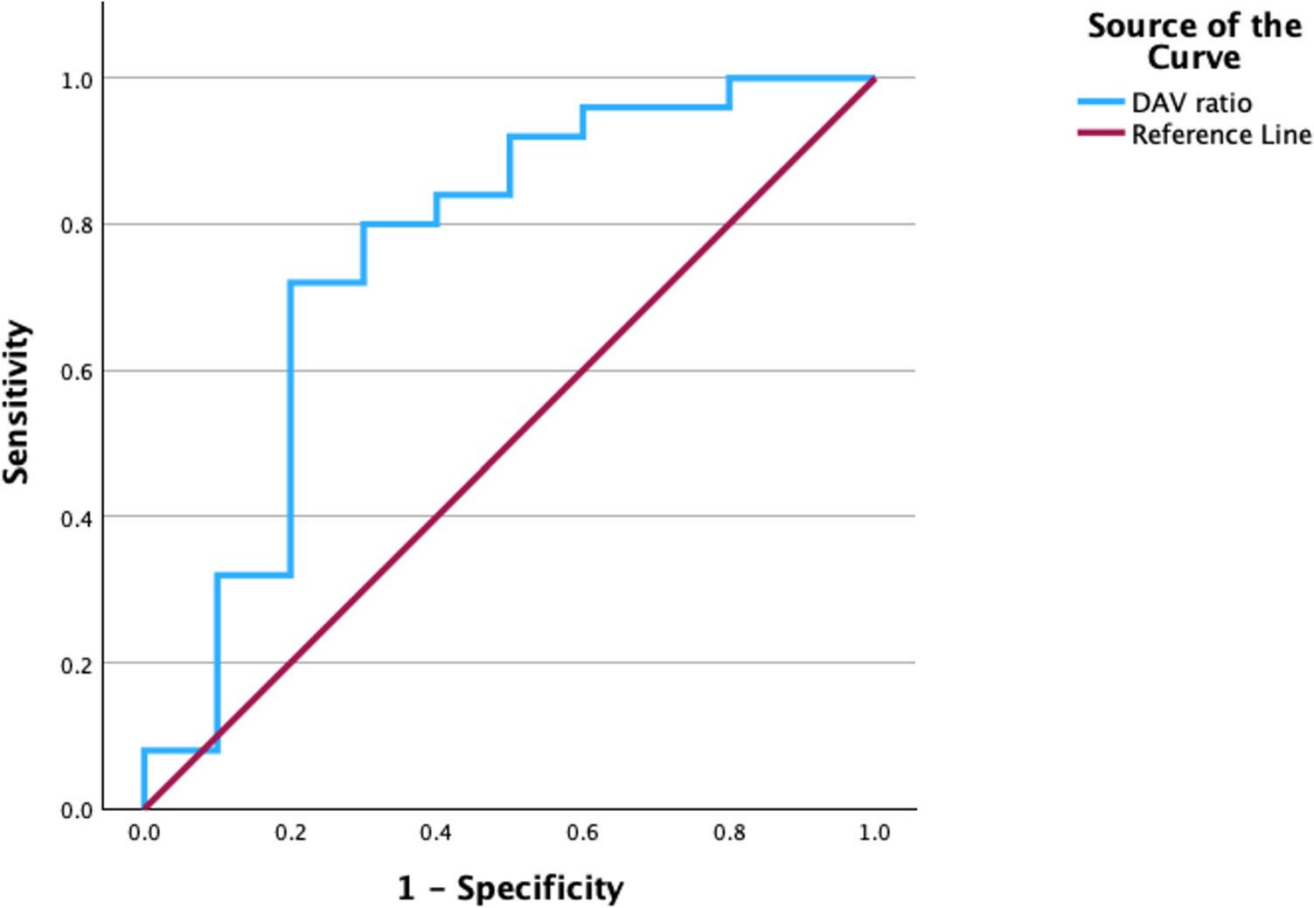
Receiver operating characteristic (ROC) curve of device-aneurysm volume (DAV) ratio to predict occlusion status. This ROC curve shows the ability of DAV ratio to predict occlusion status. Blue line, ROC curve. The area under the curve is 0.76, indicating the accuracy of the DAV ratio as a predictor, with a confidence interval (CI) of 0.56–0.96. AUC, area under receiver operating characteristics curve; CI, 95% confidence interval; ROC, receiver operating characteristics

We treated 35 patients with unruptured WNBAs using the WEB device between February 2021 and August 2024. Table 1 summarizes their baseline characteristics. The median patient age (± standard deviation; SD) was 70.0 ± 9.3 (45–76) years. The median aneurysm maximum size, neck size, height, and width were 7.5 ± 1.6, 4.3 ± 1.3 5.9 ± 1.3, and 6.3 ± 1.2 mm. 13 (37.1%), 13 (37.1%), five (14.3%), two (5.7%), one (2.9) and one (2.9%) aneurysm was respectively located in the anterior communicating, middle cerebral, basilar (top), distal anterior cerebral, posterior communicating and vertebral artery-posterior inferior cerebral arteries. The treatment success rate was 35 (100%) of 35. The WEBs in five (12.2%) patients had to be changed because of sizing errors on the first attempt. The median elapsed time to the final follow-up visit was 365 ± 184.0 days. The mean mRS score was 0 in all 35 patients (100%). One (2.9%) patient peri-operatively developed a transient ischemic attack with hemiparesis, but symptomatic complications were not permanent in any of the patients. Two (5.7%) patients required a redo procedure (Table 2). Thirty-five patients were followed-up by angiography. The primary outcomes comprised complete occlusion, categorized as WOS A and B in 19 (54.3%) and six (17.1%) patients, respectively. Seven (20.0%) and three (8.6%) patient was characterized as WOS C and D, respectively (Table 3). Table 4 summarizes the outcomes of embolization after WEB treatment. The DAV ratio significantly differed between the two groups, whereas aneurysm measurements, location, and follow-up duration did not significantly differ among the four groups. The median DAV ratio was 0.97 in the group with complete occlusion and 0.84 in the WOS C and D groups (*p* = 0.002). The AUC for the DAV ratio in the WOS A and B, and the C and D groups at follow-up was 0.76 (CI: 0.56–0.96; Fig. 3). The optimal DAV ratio cut-off for complete occlusion at follow-up was 0.90, as it had the highest Youden index.

**Table 1:** Baseline characteristics for WEB treatment

Characteristics	
Patients (*n*)	35
Median (SD) age, y	70.0 (9.3)
Female sex (*n*) Median (SD) dimensions (mm)	20 (57.1%)
*Aneurysm	7.5 (1.6)
Neck	4.3 (1.3)
Height	5.9 (1.3)
Width	6.3 (1.2)
Aneurysm location	
Anterior communication artery, *n* (%)	13 (37.1)
Middle cerebral artery, *n* (%)	13 (37.1)
Basilar artery top, *n* (%)	5 (14.3)
Distal ACA, *n* (%)	2 (5.7)
Posterior communicating artery, *n* (%)	1 (2.9)
VA-PICA, *n* (%)	1 (2.9)
Treatment success	35 (100)
WEBs exchanged, *n* (%)	5 (12.2)
Median (SD) follow-up (days)	365 (184.0)

*ACA* Anterior cerebral artery, *SD* Standard deviation, *VA-PICA* Vertebral artery-posterior inferior cerebral artery, *WEB* Woven EndoBridge

*Maximum size

**Table 2:** Clinical outcomes after WEB treatment at follow-up

Outcome	
Treated aneurysms, *n*	35
mRS 0, *n*	35 (100%)
Major stroke complications, *n*	0 (0%)
Transient ischemic attacks, *n*	1 (2.9%)
Redo treatment, *n*	2 (5.7%)

*mRS* modified Rankin scale, *WEB* Woven EndoBridge

**Table 3:** Angiographic outcomes after Woven EndoBridge treatment

Outcome	
Follow-up angiography (*n*)	35
Woven EndoBridge Occlusion Scale, *n* (%)	
A	19 (54.3%)
B	6 (17.1%)
C	7 (20.0%)
D	3 (8.6%)

**Table 4:** Outcome of occlusion at follow-up

Outcome	Complete occlusion (WOS A and B)	WOS C and D	P
Follow-up angiography (*n*)	25	10	
Age, years, median (SD)	71 (9.9)	68.5 (7.9)	0.67
Female sex, *n*	14 (56.0%)	6 (60%)	1.0
Median (SD) dimensions (mm)			
Aneurysm	7.5 (1.4)	8.1 (2.0)	0.24
Neck	4.3 (1.0)	4.6 (2.0)	0.13
Height	5.9 (1.2)	5.2 (1.5)	0.90
Width	6.0 (1.3)	6.5 (1.1)	0.19
Aneurysm location			
Anterior communication artery, *n*	8	5	0.44
Middle cerebral artery, *n*	10	3	0.71
Basilar artery top, *n*	4	1	1.0
Distal ACA, *n*	1	1	0.50
Posterior communicating artery, *n*	1	0	1.0
VA-PICA, *n*	1	0	1.0
Treatment success, *n*	25 (100%)	10 (100%)	
Median (SD) follow-up, days	365 (139.7)	365 (269.8)	0.40
Median (SD) device volume, mm^3^	151 (95.7)	134.5 (141.3)	0.28
Median (SD) aneurysm volume, mm	154 (89.4)	150.5 (159.0)	0.09
Median (SD) device-aneurysm ratio, mm^3^	0.97 (0.18)	0.84 (0.20)	0.002

*ACA* Anterior cerebral artery, *SD* Standard deviation, *VA-PICA* Vertebral artery-posterior inferior cerebellar artery, *WOS* Woven EndoBridge Occlusion Scale

## Discussion

The median DAV ratios were 0.97 in the group with complete occlusion (WOS A and B) and 0.84 in the WOS C and D group (*p* = 0.002). This study demonstrated that DAV ratios were significantly higher in patients with complete occlusion (WOS A and B) compared to those with incomplete occlusion (WOS C and D) at follow-up. The optimal DAV ratio cut-off for complete occlusion at follow-up was 0.90.

Two prospective studies have proven the safety and efficacy of WEB treatment over a period of > 5 years [9, 15] and found that rates of redo procedures after initial WEB treatment are 8.1‒15.5% over the long term [9, 13, 15]. Occlusion status at the time of redo procedures consisted of neck and aneurysmal remnants in 2 (2.1%) and 9 (9.5%) aneurysms, respectively [15]. The WEB-IT study found that complete occlusion included the WEB Occlusion Scale (WOS) grades A and B. However, 2.5% of these patients with complete occlusion in images at 1 year of follow-up required redo procedures within 5 years. About 18.2% and 33.3% of patients with WOS C and D, respectively, who were followed up by imaging at 1 year, required a redo procedure within 5 years. Residual neck and aneurysm filling after WEB treatment were predictors of redo procedures [9].

The prospective WEB-IT and WEBCAST/WEBCAST-2 studies found no adverse events related to the device after the procedure, and complete occlusion rates were 58.1% and 51.6%, with adequate rates of 87.2% and 77.9%, respectively at 5 years of follow-up. Redo procedures were required for 15.5% and 11.6% of patients [9, 15]. The Matrix and Platinum Science (MAPS) trial followed up patients for 5 years, but did not compare long-term anatomical results among the endovascular techniques in the WNBA subset. Furthermore, WNBAs were not separately analyzed and the status of aneurysm occlusion at 5 years was not reported [14]. However, stent-assisted coiling revealed worse anatomical results in a MAPS subgroup at 1 year than those found in prospective WEB studies, with complete and adequate occlusion rates of 18.6% and 45.7%, respectively [12]. The redo rate in the present study was 8%, which was lower than those in previous reports [9, 15], and shorter than the long-term rate in these studies. The aneurysm occlusion status at redo in the WEBCAST/WEBCAST2 studies was neck and aneurysmal remnants in 2 (2.1%) and 9 (9.5%) patients, respectively [15]. The WEB-IT study found that 2.5% of patients who had complete occlusion according to images at 1 year of follow-up required a redo within 5 years; 18.2% and 33.3% of patients with WOS C and D at 1 year of follow-up also required a redo at 5 years. Residual neck and aneurysm filling after WEB treatment was associated with redo procedures [9]. Therefore, comparing complete occlusion with WOS C and D in this study is reasonable. Selecting the optimal WEB size is essential for treatment that results in complete occlusion at follow-up.

The recommended WEB size is determined based on the + 1/–1 rule or the size chart provided by the manufacturer that is based on linear parameters, such as aneurysmal width and height [11]. By consistently applying WEB oversizing, the mid-term complete occlusion rate (66%) exceeded the rates in WEB-IT (58.1%) and WEBCAST/WEBCAST-2 (51.6%) [10]. Oversizing the WEB will change the shape of the device, which might lead to protrusion of the WEB base into the parent artery and favor thromboembolic insults [10]. While aneurysmal measurements are conventionally manual and recommended, software is gaining recognition as an alternative way to select the optimal WEB size.

Cagnazzo et al. described their experience of using automated software to predict the optimum WEB device by reconstructing an aneurysm using 3D rotational angiography. The automated software reduced procedural duration, radiation dose, avoided incorrect device selection, and multiple interventions [5]. Sameer et al. described the DAV ratio using automated 3D software. The highest success rate for WEB device implantation that they achieved was a DAV ratio of 0.6‒0.8 [4]. The ideal WEB-aneurysm volume (iWAVe) ratio has been introduced as a novel, volume-based parameter for optimal WEB sizing. The iWAVe ratio was determined by evaluating aneurysms treated with successful or unsuccessful WEB sizes. A decision-making process comprising aneurysm width and the iWAVe ratio, facilitated selection of the optimal WEB size. Logistic regression analysis associated an iWAVe ratio of 0.90‒1.16 with > 80% probability of sizing success, as indicated by the lower 95% CI [16]. However, these ratios are indicated only for successful implantation, and their application to predict complete occlusion after WEB treatment is not guaranteed.

We found that the DAV ratio in the groups with complete occlusion was significantly higher than that in the groups with WOS C and D during follow-up. The optimal DAV ratio cut-off for complete occlusion at follow-up was 0.90. This study suggests that oversizing the WEB device contributes to better occlusion outcomes, which may explain why this cut-off was higher than previous reports. However, oversizing can lead to the need for device replacement. In our study, the WEB had to be replaced in 5 patients (12.2%) due to sizing errors during the first attempt. Integrating conventional WEB sizing strategies (e.g., + 1 or + 2 mm oversizing and –1 or –2 mm adjustments) with a target DAV ratio of 0.90 appears crucial for optimal device selection.

While factors for morphological occlusion status in WEB treatment have been discussed, studies of recurrence and safety of the WEB device over 7 years have found less favorable occlusion rates, especially for aneurysms that are long, or have a wide neck, or a high neck-to-body ratio. Recurrent aneurysms often have thrombosed segments, complex morphology, a preprocedural diameter ≥ 5 mm, and a history of rupture [1]. The likelihood of achieving good occlusion was fivefold lower when the ostium was ≥ 4, than < 4 mm [4]. The impact of lateral compression (LC) on aneurysm occlusion and redo procedures after endovascular treatment using the WEB has been documented. The optimal proportion of LC for predicting aneurysms requiring a redo procedure was ≤ 15.6%. The rate of redo procedures was significantly higher among intracranial aneurysms with ≤ 15.6% than > 15.6% LC [2]. Our study contributes novel and important information regarding the optimal DAV ratio cut-off value for predicting occlusion status after WEB treatment determined by 3D software. The DAV ratio, which can be rapidly calculated intraoperatively, is useful for selecting the optimal WEB size. We believe that the DAV ratio aids in the optimal selection of the WEB device, thus improving occlusion status at follow-up. Several simulation software programs are now available, such as Sim&Size (Sim&Cure; Grabels, France). The use of Sim&Size for endovascular treatment of intracranial aneurysms with the Pipeline embolization device (Covidien, Irvine, California) has been shown to reduce stent length and minimize the number of devices needed [6]. Such tools—including the automated volumetric software used in our study—show promise for advancing neuroendovascular therapy in the future.

Our study has some limitations that are intrinsic to a single-center series. The data were retrospectively analyzed, the sample size was small, and selection bias was evident.

## Conclusion

The DAV ratio of the groups with complete occlusion at follow-up was significantly high, with a defined cut-off value of 0.90 between complete and incomplete occlusion. The DAV ratio determined using 3D software is an important tool with the potential to achieve complete occlusion more often in future WEB treatments.

## Data Availability

No datasets were generated or analysed during the current study.
